# Bayesian Genome-Wide Association Study of Feed Efficiency Traits in Pigs

**DOI:** 10.3390/ani16172662

**Published:** 2026-08-25

**Authors:** Sara Faggion, Valentina Bonfatti, Aurora Bergamasco, Paolo Carnier

**Affiliations:** Department of Comparative Biomedicine and Food Science, University of Padova, Viale dell’Università 16, 35020 Legnaro, Padova, Italy; sara.faggion@unipd.it (S.F.); aurora.bergamasco@studenti.unipd.it (A.B.); paolo.carnier@unipd.it (P.C.)

**Keywords:** GWAS, candidate genes, residual feed intake, feed conversion ratio, feed intake, Bayesian analysis

## Abstract

Feed efficiency traits are increasingly important in pig production for improving profitability and environmental sustainability. Understanding their genetic basis is crucial for uncovering underlying biological mechanisms and informing selection strategies. In this study, we analyzed residual feed intake (RFI), feed conversion ratio (FCR), and average daily feed intake (ADFI) in 201 animals. Three separate Bayesian GWASs were conducted using 29,844 SNPs in a case–control design, with the lowest and highest 15% of the phenotypic distribution selected as controls and cases (N = 30 per group), respectively, for each trait. The results confirmed the polygenic nature of the traits, identifying 4 SNPs for RFI on *Sus scrofa* chromosomes (SSC) 3, 13, and 15 with high posterior probability for the direction of their effects; 4 SNPs for FCR on SSC 8, 14, and 17; and 8 SNPs for ADFI on SSC 1, 2, 6, 8, and 11. A candidate gene search identified 41 potential genes involved in diverse biological processes, including feed efficiency, intestinal development, tissue remodeling and integrity, nutrient transport and absorption, metabolic homeostasis, cellular signaling, energy sensing, and neurological regulation. These genes formed a highly interconnected network, highlighting the complexity of feed efficiency and the interplay among multiple physiological, metabolic, and regulatory pathways.

## Simple Summary

In this study, we investigated the genetic basis of key feed efficiency traits, namely residual feed intake, feed conversion ratio, and average daily feed intake in a commercial line of heavy pigs. Using a Bayesian case–control genome-wide association approach, we identified, for each trait, multiple SNPs with high posterior probability for the direction of their effects, confirming that feed efficiency traits are polygenic. Potential candidate genes involved in feed efficiency, as well as in nutrient transport and absorption, metabolic homeostasis, immune functions, cellular signaling, energy sensing, neurological processes, and other processes, were identified. These findings highlight the complexity of feed efficiency and contribute to expanding current knowledge of the biological mechanisms underlying this trait in pigs.

## 1. Introduction

Maximizing feed efficiency has become one of the most important goals in pig production, offering significant benefits in terms of environmental sustainability, farm profitability, and resource use [1]. Feed efficiency is a measure of how effectively the animal utilizes nutrient resources for maintenance, growth, and development, and it is a complex trait influenced by multiple biological and environmental factors [2,3]. The most common measure of feed efficiency is feed conversion ratio (FCR), which quantifies the amount of feed consumed per unit of body weight gain. However, it is strongly associated with feed intake and growth rate [4] and its reduction by selective breeding may result in faster-growing or larger pigs not necessarily characterized by improved metabolic efficiency in nutrient utilization [5].

Residual feed intake (RFI) has emerged as a more biologically informative trait for feed efficiency. It is defined as the difference between an animal’s observed feed intake and the intake expected based on maintenance requirements and production outputs [6], typically adjusted for factors such as metabolic body weight and growth rate [7]. Consequently, RFI is independent of growth performance and body size, and by design, also statistically independent of predicted feed intake based on these traits, thereby serving as a measure of the animal’s intrinsic metabolic efficiency in nutrient utilization.

Phenotypic variation in such a complex trait can be explored through multiple omics layers, enabling the identification of molecular markers associated with trait expression, as well as the underlying functional pathways and regulatory networks involved in their biological regulation [8]. Genome-wide association studies (GWASs) have been widely used to detect associations between genetic variants and quantitative traits, resulting in the mapping of over 57,000 quantitative trait loci (QTLs) to approximately 400 distinct traits in the swine genome (Pig QTL Database; https://www.animalgenome.org/ accessed on 13 March 2026) [9]. Among these, numerous genomic regions, each exerting a small effect, have been identified as significantly associated with feed efficiency traits and are distributed across nearly all *Sus scrofa* chromosomes (SSCs), highlighting the polygenic nature of these traits. However, the genomic locations of these regions vary depending on factors such as breed, age, and body weight range [10,11,12].

Investigations into the biological pathways underlying feed efficiency traits have identified a range of biological processes, including lipid and carbohydrate metabolism, growth factor signaling, insulin secretion regulation, and immune response [11]. However, a consistent feature across these studies is that pigs were typically from lines or breeds selected for lean growth and tested between 25 and 120 kg body weight, reflecting standard commercial practices prevalent in global pig production systems.

In contrast, the Italian pig breeding industry primarily focuses on producing heavy pigs for high-quality Protected Designation of Origin (PDO) dry-cured hams, where pigs are raised until they reach approximately 160–200 kg body weight and 9 months of age to meet the stringent quality standards imposed by product specifications [13]. This results in animals that are both heavier and fatter compared to those in other parts of the world. Selection programs in this context are tailored for carcass quality and suitability for dry-curing, rather than lean growth. Because RFI has never been studied in heavy pig populations, current knowledge of the genetic basis of feed efficiency may not be transferable to this production system. Furthermore, the cost and logistical complexity of measuring individual feed intake in large populations of heavy pigs limit the feasibility of routine RFI-based selection. These challenges highlight the need for genomic tools that can assist in selecting for metabolic efficiency without relying on extensive phenotyping.

In this study, we addressed the limited knowledge of the genetic architecture underlying feed efficiency traits in a purebred heavy pig line selected for dry-cured ham production. Specifically, we investigated RFI, FCR, and average daily feed intake (ADFI) by performing a genome-wide association study using a Bayesian framework combined with an extreme phenotype strategy. Subsequent post-GWAS analyses included the identification of potential candidate genes, and integrative gene network analyses aimed at elucidating possible functional pathways and molecular mechanisms underlying the observed phenotypic variation in the studied traits. To our knowledge, this is the first study investigating the genomic architecture of feed efficiency in heavy pigs, providing valuable insights to guide genomic selection strategies adapted to the unique demands of high-quality dry-cured ham production.

## 2. Materials and Methods

### 2.1. Animal Population and Experimental Design

A total of 207 purebred Goland C21 (Gorzagri, Fonzaso, Italy) pigs (101 gilts and 106 barrows), offspring of 23 sires and 112 dams, were used in the experimental trial. The pigs were raised in 4 consecutive rearing batches (N = 48 to 55 pigs each) on a single farm and fed identical commercial diets. At 148 ± 1 days of age and an average body weight (BW) of 93.6 ± 8.8 kg, the pigs of the 4 batches were transferred sequentially to the experimental station at the University of Padova (Legnaro, Italy), between June 2018 and July 2020. Pigs from each batch were randomly assigned to 4 pens and fed a high-protein diet ad libitum, with no limitations on indispensable amino acid content. In each pen, individual feed intake was monitored by a single-space electronic feeder (Compident Pig–MLP, Schauer Agrotronic, Prambachkirchen, Austria). Further details on the experimental design, ingredients and nutrient content of the diets are described in [14,15]. The experimental period lasted 67 days, and the trial ended upon pigs reaching the target slaughter weight (170 kg body weight). Throughout the experimental period, individual BW (kg) of each pig was measured approximately every two weeks (8.8 ± 1.54 observations per pig), whereas individual backfat thickness (BF, mm) was assessed less frequently (5.1 ± 1.26 measures per pig) [14]. Individual feed intake, BW and BF were measured to obtain the data required to compute individual ADFI, RFI and FCR. The pig was the experimental unit.

### 2.2. Computation of Average Daily Feed Intake, Residual Feed Intake and Feed Conversion Ratio

Individual ADFI (kg/day) was calculated by dividing the total feed intake recorded by electronic feeders over the experimental period by the number of test days (N = 67). Individual empty BW (EBW, kg), body lipid mass (BL, kg) and body protein mass (BP, kg) were estimated from BW and BF using the following equations developed by [16,17]:

(1) $$\mathrm{EBW}=0.914\;\times {\;\mathrm{BW}}^{1.008}$$

(2) $$\mathrm{BL}=\frac{9.17+\left(0.7\times \mathrm{BF}\right)}{100}\times \mathrm{BW}$$

(3) $$\mathrm{BP}=0.1353\times {\left(\mathrm{EBW}-\mathrm{BL}\right)}^{1.1175}$$

For each pig, the initial and final EBW, BL, and BP during the experimental period were derived from individual U-Gompertz growth curves [14]. These values were then used to compute the average daily gain in BL (BL_ADG_, kg/day), BP (BP_ADG_, kg/day), EBW (EBW_ADG_, kg/day) and the EBW at the midpoint of the experimental period (EBW_MID_, kg).

Residual feed intake (RFI, kg/day) was computed as the residual term *e* of the following linear model, as explained in [14]:

(4) $${\mathrm{ADFI}}_{\mathrm{ij}}=\;\mu \;+{\mathrm{sex}}_{i}+{\;\beta }_{1}{\mathrm{EBW}}_{{\mathrm{MID}}_{\mathrm{ij}}}\;+\beta_{2}{\mathrm{BL}}_{{\mathrm{ADG}}_{\mathrm{ij}}}+{\;\beta }_{3}{\mathrm{BP}}_{{\mathrm{ADG}}_{\mathrm{ij}}}+{\;e}_{\mathrm{ij}}$$

where ADFI was the individual ADFI (kg/day) of animal *j*, µ was the overall intercept of the model, sex was the fixed effect of sex *i* with 2 levels (*i* = 1: gilts, *i* = 2: barrows), EBW_MID_, BL_ADG_, BP_ADG_ had the same meaning as before, and β_1_, β_2_, and β_3_ were the linear regression coefficients of ADFI on EBW_MID_, BL_ADG_ and BP_ADG_, respectively. The feed conversion ratio (FCR, kg/kg) was calculated as the ratio of ADFI to EBW_ADG_.

### 2.3. Genotyping

DNA was extracted from saliva samples collected from each of the 207 pigs included in the experiment (GeneSeek Inc. in Lincoln, NE, USA). Genotyping was performed using the high-density GGP Porcine 50K single nucleotide polymorphism (SNP) chip, yielding successful genotypes for 201 pigs. SNP quality control was performed using a custom R script [18], excluding markers with a minor allele frequency lower than 1% and a call rate lower than 95%. Missing genotypes were imputed using the FImpute software (version 2.2; [19]), leveraging genotype data and pedigree information from the Goland C21 pig line. After filtering and imputation, a total of 29,844 SNPs were retained for downstream analyses.

### 2.4. Genome-Wide Association Studies

The genome-wide association analyses for RFI, ADFI and FCR were performed using a case–control design, following the two-step methodology proposed by [20] and employing a Bayesian approach. Phenotypes were converted into binary traits by assigning animals in the lowest 15% of the phenotypic distribution to class 0 (N = 30) and those in the highest 15% to class 1 (N = 30). For each trait, individuals were assigned to the extreme phenotype groups based on the raw phenotypic values. For RFI, this corresponded to the residual obtained from model (4). This approach was selected to address the limited sample size and enhance statistical power by contrasting individuals at the extremes of the phenotypic distribution [21,22,23,24,25,26]. By focusing on animals with divergent phenotypes, this approach increases the likelihood of identifying genetic variants with meaningful contributions to the traits of interest, as individuals at the tails of the distribution are more likely to carry alleles with larger effects, facilitating the detection of genotype–phenotype associations. Moreover, excluding animals with intermediate phenotypes might help reduce background noise [27].

Step 1 consisted of a preliminary SNP scan to identify a set of potentially significant markers. This step was carried out by computing Bayes factors for each SNP, comparing a baseline logit model with a full logit model. The baseline model had the following form:

(5) $$log\left(\frac{P\left(y_{i}=1\right)}{P\left(y_{i}=0\right)}\right)=\beta_{0}+\;\overset{K}{\underset{k=1}{\sum }}\beta_{k}x_{ki}$$

where $P\left(y_{i}=1\right)$ and $P\left(y_{i}=0\right)$ were the probabilities of individual *i* belonging to class 1 (high phenotypic value) or class 0 (low phenotypic value), respectively, $\beta_{0}$ was the intercept, $\beta_{k}$ was the regression coefficient associated with the *k*-th dummy variable $x_{ki}$, where batch was represented by 3 indicator variables (batch 1 was the reference) and sex by one indicator variable (sex 1 corresponding to gilts was the reference). For RFI, only batch effect was included, whereas for ADFI and FCR, both batch and sex effects were included.

The model was defined by the likelihood $y_{i}\;\sim \;Bernoulli\;\left(p_{i}\right)$, where $p_{i}=\;P\left(y_{i}=1\right)$; as recommended by [20], for Bayes factor estimation, vague independent normal priors were used for intercept (N(0, 5)) and regression coefficients (N(0, 1)). For each model, 2 chains were run, with 2000 iterations each, 500 warm-up iterations, and a thinning interval of 10. The analyses were performed using the R package brms v. 2.23.0 [28,29] employing Hamiltonian Monte Carlo chains and No-U-Turn sampler. Model convergence and fit were assessed using both the built-in convergence diagnostics provided by the brms package ($\;\hat{R}$, bulk effective sample size, tail effective sample size) and visual diagnostic tools available in the R package bayesplot v. 1.15.0 (posterior predictive checks; [30]).

For each independent SNP analysis, the full logit model followed the same structure as model (5), retaining batch (and sex, for ADFI and FCR) as fixed effects, with the addition of the SNP genotype coded as number of copies of the minor allele (0, 1 or 2). Plots of the log-transformed Bayes factors obtained for all SNPs were generated using ggplot2 v. 4.0.1 [31].

The 50 SNPs characterized by the highest Bayes factors were retained for Step 2, where a logit model was built to jointly model the effects of batch, sex and all the selected SNPs on the binary phenotype as follows:

(6) $$log\left(\frac{P\left(y_{i}=1\right)}{P\left(y_{i}=0\right)}\right)=\beta_{0}+\;\overset{K}{\underset{k=1}{\sum }}\beta_{k}x_{ki}+\;\overset{50}{\underset{j=1}{\sum }}\alpha_{j}z_{ji}$$

where $\alpha_{j}$ was the regression coefficient for the *j*-th SNP and $z_{ji}$ was the genotype code (0, 1, or 2 copies of the minor allele) for individual *i* at SNP *j*, and all other terms had the same meaning as in model (5). The likelihood and prior distributions were the same as before; 4 chains were run, with 10,000 iterations each, 2000 warm-up iterations, and a thinning interval of 1. As in Step 1, all analyses were conducted in R using the brms package v. 2.23.0 [28,29] and model performance was evaluated through the same convergence diagnostics.

For each SNP, the posterior samples of the regression coefficients were extracted, and the posterior mean and 95% highest posterior density interval (HPD95%) of the regression coefficient were computed using the R package boa v. 1.1.8-2 [32]. Odds ratios (ORs) were obtained by exponentiating the posterior samples of the regression coefficients, and the probability of an OR (P_OR_) being greater than 1 (if the mean OR was greater than 1) or lower than 1 (if the mean OR was lower than 1) was calculated. SNPs with a corresponding P_OR_ greater than 0.85 were considered to show stronger evidence of an effect on the phenotype and were retained for candidate gene search.

### 2.5. Candidate Gene Search and Gene Network Analysis

Candidate genes within 0.2 Mb upstream and downstream of the coordinates of the identified SNPs were identified using the Ensembl VEP (variant effect predictor) tool (https://www.ensembl.org/info/docs/tools/vep/index.html; accessed on 10 May 2026) and gene annotation information from the *Sus scrofa* reference genome (Sscrofa11.1). The Database for Annotation, Visualization, and Integrated Discovery (DAVID) [33] was used to retrieve the biological meaning and pathways associated with the identified genes. To explore potential interactions among the identified genes, including protein–protein and genetic interactions, shared pathways, co-expression, co-localization, and similarities in protein domains, a gene network analysis was conducted using the GeneMANIA plug-in [34] within the Cytoscape platform [35]. The plug-in integrates available information from a wide range of sources, from individual studies to large databases of functional interaction networks [34].

## 3. Results and Discussion

### 3.1. Descriptive Statistics of Feed Efficiency Traits

Descriptive statistics for all traits measured in the experimental animals (BW, EBW, BL, BP, EBW_MID_, EBW_ADG_, BL_ADG_ and BP_ADG_), which were used to derive the traits analyzed in the present study, were reported in detail in a previous publication [14].

Descriptive statistics of the traits included in the current study are presented in Table 1. Pigs exhibited considerable variability in RFI (SD = 0.18 kg/day). This pattern may be explained by the genetic background of the experimental pigs, which originated from boars belonging to a sire line primarily selected for improved ham quality rather than feed efficiency traits. Considerable variability was also observed in ADFI (SD = 0.47 kg/day) and in FCR (SD = 0.30 kg/kg).

The phenotypic extremes for RFI, ADFI and FCR, defined as the lowest and highest 15% of the distribution, showed substantial divergence, supporting the suitability of this grouping criterion for a case–control GWAS, a strategy also adopted in previous studies in pigs [36] and other species [22,24,25], and highlighting its potential to capture genetic contrasts. The largest difference was observed for RFI (194.77% difference between the means of the low and high groups). For ADFI and FCR, differences of 58.30% and 26.79% were observed, respectively.

### 3.2. Genomic Regions Associated with Feed Efficiency Traits

Despite the relatively small number of pigs included in the study, mainly due to the experimental and logistical challenges of measuring individual feed intake in large populations of heavy pigs, Bayesian GWASs following a case/control strategy enabled the identification of SNPs with evidence of an effect on the traits under investigation.

Several studies have demonstrated the advantages of focusing on the tails of the phenotypic distribution using individuals with extremely high or low phenotypic values, rather than analyzing the trait as a continuous variable. The rationale behind this strategy is that individuals at the extremes of the phenotypic distribution are more likely to carry genetic variants with large effects. Consequently, concentrating on these individuals increases the likelihood of detecting variants that contribute substantially to phenotypic variation, an approach that has been widely applied in studies investigating rare genetic variants [21,22,23,24,25,26]. In studies on quantitative traits in humans, Refs. [22,24] adopted an approach similar to that used in the present study. Specifically, they selected individuals from the upper and lower 10% of the phenotypic distribution and dichotomized them into cases and controls, demonstrating that this strategy was more effective at detecting associations than linear regression analysis based on the complete phenotypic distribution, without loss of information. In [24], the selection thresholds were progressively widened to include the upper and lower 20% and 30% of the phenotypic distribution. Although this increased the sample size, shifting the cut-points towards the median introduced relatively uninformative observations, thereby diluting the contrast between groups and reducing statistical power. Also, Refs. [23,25] further demonstrated that, when risk genotypes are rare in the population and have relatively small effects, extreme phenotype sampling is advantageous. They showed that selecting individuals from the tails of the phenotypic distribution in a balanced case–control design requires a smaller sample size than a quantitative trait analysis to achieve equivalent statistical power. The models proposed in those studies, originally developed for relatively rare diseases, can also be applied to the rationale underlying the extreme phenotype approach for continuous traits across a range of quantitative traits and species.

**Figure 1:**
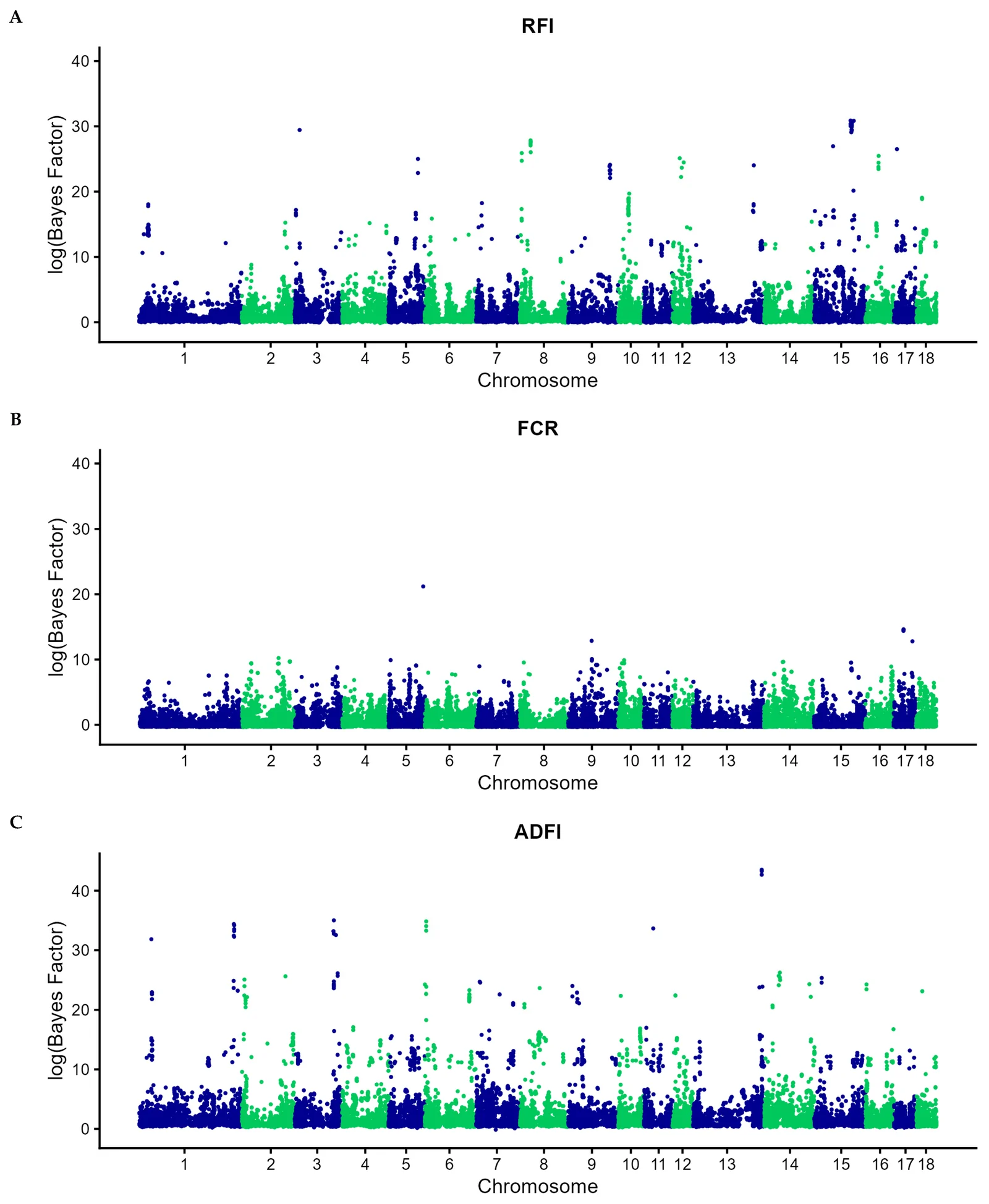
Log-transformed Bayes factors obtained in the case-control Bayesian GWAS for each SNP: (**A**) residual feed intake (RFI), (**B**) feed conversion ratio (FCR), and (**C**) average daily feed intake (ADFI).

The plots of the log-transformed Bayes factors for RFI, ADFI, and FCR are reported in Figure 1. Log-transformed Bayes factors ranged from −0.16 to 30.88 for RFI, from −0.37 to 21.20 for FCR, and from −0.14 to 43.53 for ADFI. Among the top 50 ranked SNPs, log(Bayes factor) values exceeded 18.84 for RFI, 8.46 for FCR, and 23.20 for ADFI. Bayes factors are used to compare two competing models: a null model, in which all markers are assumed to have no effect, and an alternative model, in which at least one marker contributes to the trait. Thus, the Bayes factor quantifies the evidence supporting the presence or absence of genetic variation attributable to the genomic region under investigation. When evidence is combined across neighboring markers, the support for either model is strengthened, allowing association signals to be captured more effectively. Bayes factors were effectively used as a marker evaluation method or for QTL mapping, demonstrating consistent results across traits and species [37,38,39,40,41,42]. In our manuscript, as suggested by [20] and [43], Bayes factors were used to rank SNPs in terms of association and select those that should be retained for the subsequent phase, where selected markers were fitted simultaneously.

For RFI, GWAS results are reported in Table 2 and Figure S1. The analysis evidenced a signal on SSC15, where two SNPs were identified with P_OR_ of 0.85 and 0.99. Additionally, relevant signals were observed on SSC3 (P_OR_ = 0.94) and on SSC13 (P_OR_ = 0.97; Table 2; Figure S1). Among these associations, the strongest evidence was observed for the SNP on SSC15 located at 57,638,216 bp, which showed both a high posterior probability and a confidence interval of the OR excluding 1. The remaining SNPs, although characterized by OR confidence intervals including 1, still exhibited posterior P_OR_ of effect direction ranging between 0.85 and 0.97, supporting the consistency of the inferred associations despite the uncertainty reflected by the wide intervals. Overall, these findings support the involvement of SSC15 in RFI variation, while also highlighting a potential contribution of the region identified on SSC3 and SSC13.

Bayesian GWAS does not classify SNPs as significant or non-significant; it quantifies the posterior probability of an association or, as reported in the current manuscript, the posterior probability of the direction of the SNP effect, rather than simply the probability that an association exists. In our analysis, SNPs with a P_OR_ greater than 0.85 were retained for subsequent candidate gene search analysis. It is important to note that the P_OR_ is itself a continuous probability derived from the posterior distribution of SNP effects, representing the probability that the effect is in the estimated direction given the observed data and the specified prior distribution. Consequently, unlike a frequentist significance threshold, a P_OR_ cutoff is not a formal decision criterion separating “significant” from “non-significant” associations. Rather, it is an arbitrary reporting criterion used to highlight SNPs supported by stronger posterior evidence. In the literature, probability values of the direction of the SNP effect greater than 0.75 were used to retain and interpret the results [44]. In general, in Bayesian regression models, there is no universally accepted criterion for defining the “relevance” of an association, nor well-established thresholds for determining it [42]. Several criteria have been proposed, including maximum a posteriori estimates [20], the proportion of additive genetic variance explained [45], and the already mentioned Bayes factors [37,38,39,40,41,42]. However, the thresholds used to report or highlight associations are generally study-specific and depend on the analytical context. The interpretation of P_OR_ should also be considered together with the statistical framework of Bayesian GWAS. It is also worth noting that focusing on chromosome-level signals rather than individual SNPs is more appropriate, as the statistical framework underlying Bayesian GWAS differs fundamentally from that of traditional single-SNP GWAS. Traditional single-SNP approaches detect QTL only when at least one SNP is in linkage disequilibrium with the causal SNP across the analyzed population; thus, the effects of significant markers tend to be overestimated (“Beavis effect”) [46]. Conventional multiple-testing corrections, such as the Bonferroni correction, treat markers as independent tests despite linkage disequilibrium among neighboring SNPs, resulting in conservative significance thresholds and reduced statistical power. Bayesian approaches estimate the effects of all SNPs simultaneously under a specified prior distribution of marker effects. Consequently, the association signal is not necessarily attributed to a single SNP but can be distributed across multiple markers in linkage disequilibrium with the causal variant. This feature is particularly advantageous when the causal variant itself is not included in the genotyped marker panel, as is typically the case with SNP arrays. In such situations, groups or windows of SNPs surrounding the causal locus may capture the association signal more effectively than individual markers [47]. For this reason, chromosome-level or regional signals may be biologically more informative than individual SNP associations.

While the current study identified three main chromosomes associated with RFI, previous research has highlighted the polygenic and complex nature of this trait. Indeed, several studies have reported significant associations between SNPs and RFI across a wide range of chromosomes in different pig breeds, including: SSC1, 2, 3, 7, 8, 9, 10, 14, 15, and 17 in Yorkshire pigs [48,49,50]; SSC1, 7, 8, 9, and 13 in Duroc pigs [50,51,52]; SSC1, 2, 10, 12, 13, and 15 in Junmu No. 1 pigs [36]; SSC9 in crossbred (Landrace × Yorkshire) × Duroc pigs [3]; and SSC1, 2, 3, 4, 6, 8, 9, 10, and 11 in Landrace pigs [53].

Factors such as sample size, breed, age, body weight range, and the model used to estimate RFI appeared to substantially influence GWAS results. In the present study, RFI was estimated using a model that accounted for animal sex, average empty body weight (EBW), and tissue accretion (daily gains in body lipid, BL_ADG_, and body protein, BP_ADG_). By accounting for these factors, the model adjusted feed intake for variation attributable to maintenance requirements and to the differing energetic costs associated with lean and fat tissue deposition, thereby providing a measure of feed efficiency that is independent of growth and body composition, potentially explaining the lower number of chromosomes found to be associated with the trait. Additionally, the GWAS methodology itself may influence the results obtained. Bayesian GWAS approaches differ fundamentally from traditional single-marker GWAS because they estimate all marker effects jointly under a prior distribution. Compared with traditional single-SNP analyses, this framework provides an alternative way of accounting for multiple testing, marker-effect shrinkage, false discovery control and, to some extent, population structure [37,42,54,55]. In Bayesian GWAS, false-positive findings can be controlled by specifying an acceptable proportion of false discoveries among the reported associations through posterior probabilities. Unlike frequentist multiple-testing corrections, Bayesian approaches do not require increasingly stringent significance thresholds as the number of markers increases. Consequently, the power to detect true associations is not intrinsically reduced by testing a larger number of markers [54]. By fitting multiple SNPs simultaneously, as in Step 2 where the 50 SNPs selected in Step 1 were jointly included in the model, estimated marker effects are already shrunk according to their prior distributions, thereby avoiding the need for post hoc multiple-testing corrections such as Bonferroni adjustment. For continuous traits, the samples retained after burn-in can be used to estimate the posterior probability of association for each SNP, defined as the proportion of iterations in which the SNP is assigned a non-zero effect in the model; posterior probabilities provide a framework for identifying association signals while controlling the posterior type I error rate [56]. Unlike p-values, posterior probabilities are themselves probabilities and therefore provide a continuous measure of the posterior evidence supporting an association. This is conceptually analogous to the probability that the OR is greater than 1 when the posterior mean OR exceeds 1, or less than 1 when the posterior mean OR is below 1, as reported in our manuscript for a binary trait. Joint estimation of multiple marker effects also reduces confounding due to population structure compared with single-marker analyses, even when population structure is not explicitly modeled. This approach has been shown to provide greater power to detect QTL than traditional single-SNP GWAS models that include population structure or breed composition effects, without increasing the false-positive rate [55,57,58].

For FCR and ADFI, details on the identified SNPs are reported in Table 3 and Table 4 and Figures S2 and S3, respectively. The SNPs identified for FCR were located on SSC8, SSC14, and SSC17 and showed posterior probabilities of effect direction ranging from 0.85 to 0.99, indicating moderate to strong support for the inferred associations (Table 3; Figure S2). The strongest signal was observed for the locus on SSC17 (position 57,705,289 bp), which displayed the highest posterior P_OR_ (0.99) and was also characterized by a confidence interval excluding 1, providing robust statistical support for the direction of the SNP effect. The remaining loci on SSC8 and SSC14 exhibited P_OR_ between 0.85 and 0.91. Although their confidence intervals included 1, indicating greater uncertainty in the estimated effects, the relatively high posterior probabilities still suggested consistent evidence of association. In particular, the two SNPs identified on SSC14 showed similar P_OR_ values and comparable levels of uncertainty. Overall, the high posterior P_OR_ supported the involvement of these genomic regions in FCR variation and provided strong evidence for the direction and magnitude of SNP effects, in particular at the SSC17 locus. These findings suggested that multiple genomic regions contribute to variation in FCR, consistent with previous studies reporting SNPs located on SSC4, SSC6, SSC7, SSC8, SSC17, and SSC18 [53,59,60]. In particular, the strong QTL identified on SSC17 by [53] in the Landrace pig breed is consistent with our results.

For ADFI, a total of 8 SNPs with moderate to high P_OR_ ranging from 0.86 to 0.98 were identified (Table 4; Figure S3), indicating consistent support for the direction of the SNP effects. Most were located on SSC1 (3 SNPs) and SSC6 (2 SNPs), with additional signals on SSC2 (1), SSC8 (1), and SSC11 (1). The strongest evidence was observed for the loci on SSC1 (position 289,692,169 bp) and SSC8 (position 60,120,775 bp), which both displayed the highest posterior P_OR_ values (0.98) and were also characterized by confidence intervals excluding 1. The remaining loci showed confidence intervals including 1, reflecting greater uncertainty in the magnitude of the estimated effects despite the relatively high posterior probabilities. In particular, the SNPs on SSC1 (excluding the one previously described), SSC2, SSC6, and SSC11 exhibited P_OR_ ranging from 0.86 to 0.97, suggesting a consistent direction of association even though the corresponding confidence intervals were wide. Overall, the combination of high posterior probabilities and, in some cases, confidence intervals excluding 1 supported the relevance of these genomic regions to ADFI. According to previous studies, ADFI was recognized as a polygenic trait, influenced by numerous SNPs, each exerting a small effect on the trait, and GWAS outcomes are highly dependent on the breed. Significant associations have been reported across multiple chromosomes: SSC1, 3, 4, 5, 6, 7, 9, 10, 14, 16, 17, and 18 in Duroc pigs [50,51,52,61]; SSC1, 3, 4, 6, 12, 13, 14, and 17 in Yorkshire pigs [48,50]; SSC1 in crossbred pigs [synthetic line × (Landrace × Large White)] [62]; and SSC2, 4, 6, 8, 9, and 12 in Landrace pigs [53].

When comparing the three traits, no overlapping genomic regions were identified across two or more traits. Notably, the absence of shared signals between RFI and FCR suggests that, despite both being commonly used indicators of feed efficiency, the two traits are only partially related at the genetic level and may therefore capture different components of feed efficiency. Consistent with this interpretation, previous studies in light pigs (maximum body weight of 115 kg) have reported only moderate genetic correlations between FCR and RFI, with estimates ranging from 0.45 to 0.70 [63,64,65]. The partial overlap between these traits likely reflects their different biological foundations. FCR directly relates feed intake to body weight gain and is therefore strongly influenced by growth rate, metabolism, body composition, and activity. In contrast, RFI is intended to measure feed efficiency independently of production traits, as it quantifies deviations between observed and expected feed intake requirements. The lack of overlap therefore highlights the complementary biological information provided by RFI and FCR and suggests that selection based on one trait may not necessarily capture the genetic determinants underlying the other.

### 3.3. Candidate Genes for RFI, FCR and ADFI

A total of 15, 10 and 16 potential candidate genes were identified for RFI, FCR and ADFI, respectively (Table 5, Table 6 and Table 7). These genes pointed to three key biological processes contributing to variation in feed efficiency and feed intake: (1) the maintenance and functional regulation of the intestinal epithelium, including epithelial differentiation, homeostasis, cellular turnover, and digestive secretory activity; (2) nutrient metabolism and energy balance pathways; (3) central nervous system pathways involved in the regulation of appetite, feed intake, and satiety signaling. Despite the absence of shared genomic regions and common candidate genes between RFI and FCR, the underlying biological functions appeared consistent. In both traits, a comparable number of candidate genes were associated with intestinal epithelial development and integrity, metabolic homeostasis, and neurological functions, suggesting that distinct genetic determinants may converge on similar physiological and regulatory mechanisms. Feed intake was largely dominated by neuronal or neurodevelopmental functions, further emphasizing the central role of the nervous system in regulating feeding behavior, appetite control and nutrient-seeking responses, ultimately contributing to differences in voluntary feed intake among individuals. Genes involved in metabolic regulation and nutrient flux were identified across all traits under investigation, suggesting the existence of common biological pathways underlying both feed intake and feed efficiency. Although different genes were implicated for each trait, their functions converged on processes related to nutrient metabolism, energy utilization, and metabolic homeostasis.

A prominent cluster of candidate genes is involved, through their encoded proteins, in actin cytoskeleton organization and cell and tissue structural remodeling, in particular related to the intestinal epithelium. In fact, the intestinal epithelium plays a central role in nutrient digestion, absorption, and barrier function, thereby directly influencing the efficiency with which animals utilize feed. Enhanced epithelial integrity and transport capacity can improve nutrient uptake and metabolic efficiency, ultimately contributing to reduced feed intake requirements and improved feed efficiency. *ARPC2* (identified for RFI) encodes an essential component of the Arp2/3 complex, which is a regulator of intestinal epithelial homeostasis and integrity, ensuring tight junction stability [66]. *PROM1* (FCR) encodes a membrane glycoprotein that is a defining marker of intestinal epithelial stem cells sustaining the high turnover of the absorptive epithelium [67]. Variation in *PROM1* expression or function could alter the renewal rate of the absorptive epithelium, with consequences for the efficiency of nutrient absorption and the energetic cost of tissue maintenance. Also implicated in the intestinal epithelium is *VIL1* (RFI), which crosslinks actin filaments within the microvilli of enterocytes and is a defining marker of intestinal epithelial differentiation [68]. Altered villin expression could affect microvilli morphology and gut absorptive capacity. *VIL1* also functions as a metabolic sensor in intestinal epithelium, as villin-deficient mice displayed altered nutrient absorption and epithelial stress responses [68]. *SPIRE2* (ADFI) is a WH2-domain actin nucleator that acts in concert with formin proteins to generate unbranched actin filaments [69]. *SPIRE2* has roles in vesicle trafficking and organelle positioning in epithelial cells, potentially affecting secretory function in the digestive tract. *TNS1* (RFI) belongs to the tensin family, involved in cell migration, mechanosensing, and tissue remodeling [70]. In Puławska and Polish Landrace *TNS1* has also been implicated in pork quality, growth performance, fat and meat carcass contents [71]. *AAMP* (RFI) is involved in endothelial tube formation and endothelial cell migration, as well as smooth muscle cell migration [72]. Smooth muscle is the type of muscle found in the walls of the gastrointestinal tract and is responsible for stirring the contents of the gut and moving food, water, and waste through the tubular chambers [73]. *TAPT1* (FCR) is a critical mediator of intracellular trafficking and primary cilium formation and plays an important role in Bone Morphogenetic Protein (BMP) signaling. Through its involvement in BMP-dependent processes, *TAPT1* contributes to the regulation of neuroendocrine functions and the maintenance of homeostasis in digestive endocrine cells [74]. In Duroc pigs, this gene is expressed in the liver and skeletal muscle, where it has been implicated in the regulation of carcass quality [75].

*RBM38* (FCR) encodes an RNA-binding protein involved in the regulation of cell proliferation, apoptosis, and mRNA processing. Its paralog, RBM24, has been associated with myogenic regulatory pathways in the Brazilian Piau pig breed [76].

A substantial number of candidate genes are associated with membrane transporters or proteins that regulate metabolism and nutrient flux. *PLEKHB2*, encoding a protein primarily responsible for mediating retrograde membrane trafficking, regulating transport and potentially influencing cell differentiation, was identified for RFI and has previously been associated with this trait in beef cattle [77]. *SLC11A1* (RFI) transports iron, manganese, and zinc out of the phagolysosomal compartment, regulating intracellular metal availability. Iron homeostasis is tightly coupled to mitochondrial function and oxidative metabolism, and variation in *SLC11A1* could alter the efficiency of energy extraction from feed substrates. This gene also has a well-established role in innate immune resistance to intracellular pathogens [78]. *SLC35F3* (FCR) is associated with thiamine transport, which is crucial for cellular glucose metabolism and energy production [79]. It is also essential for carbohydrate metabolism and acts as a key player in cellular energy pathways. *CTDSP1* (RFI) regulates the global transcriptional output of metabolically responsive gene networks by controlling the phosphorylation state of RNA polymerase II [80]. *TCF25* (ADFI) is a basic helix–loop–helix transcription factor expressed in multiple tissues, including the gastrointestinal tract and liver. Recently, its encoded protein was shown to enhance lysosomal acidification by targeting V-ATPase in response to glucose starvation, a mechanism required for energy maintenance through the promotion of catabolism under low-glucose conditions [81]. *GPBAR1* (RFI) is a membrane receptor for conjugated and unconjugated bile acids expressed in enteroendocrine L-cells, brown adipose tissue, and hypothalamic neurons [82]. Upon activation by intestinal bile acids, *GPBAR1* stimulates GLP-1 secretion from L-cells, promotes thyroid hormone activation in brown adipose tissue, and increases energy expenditure. *MC1R* (ADFI) encodes a G protein-coupled receptor for alpha-melanocyte-stimulating hormone [83]. While *MC1R* is best known for its role in pigmentation, in general the melanocortin system is a master regulator of energy homeostasis, appetite, feed intake and gut functions [84]. *CDC42SE2* (ADFI) encodes a downstream effector of the small GTPase CDC42. CDC42 signaling in enteroendocrine cells modulates the secretion of gastrointestinal hormones, which are key satiety signals [85]. Variation in *CDC42SE2* could therefore affect postprandial hormone secretion profiles and thereby influence daily feed intake. *RAE1* (FCR) mediates the nuclear export of poly(A) mRNAs encoding metabolic enzymes and regulatory factors [86]. Variation in *RAE1* activity could alter the cytoplasmic abundance of transcripts critical for hepatic lipid metabolism and intestinal secretory function. *TMBIM1* (RFI) is involved in metabolic homeostasis, and its lower expression promotes adipocyte hyperplasia and improves obesity-related metabolic diseases [87]. *RUFY4* (RFI) contains lipid-binding domains characteristic of endosomal regulators influencing the trafficking of nutrient transporters and digestive enzyme receptors in intestinal epithelial cells [88]. *VPS9D1* (ADFI) encodes a guanine nucleotide exchange factor for Rab5 GTPases, which are master regulators of early endosome fusion and recycling [89]. Rab5-GEF activity controls the rate of receptor internalization and recycling, including that of hormone receptors relevant to satiety signaling. *ST6GALNAC3* (ADFI) catalyzes the addition of sialic acid to O-glycans on cell-surface and secreted glycoproteins [90]. Sialylation of mucins and digestive enzymes affects their stability, activity, and interactions with the gut microbiome. *EGLN1* (FCR) is a central regulator of the hypoxic response, controlling the expression of hundreds of genes involved in glycolysis, angiogenesis, erythropoiesis, and mitochondrial biogenesis [91]. Variation in *EGLN1* activity could alter the threshold at which cells switch between oxidative and glycolytic metabolism, directly affecting feed conversion efficiency in tissues with high metabolic demand such as skeletal muscle. *TSNAX* (FCR) forms a complex with translin (TSN) regulating the expression of metabolic genes in the hypothalamus and has been implicated in the central regulation of body weight [92]. *LDB2* (FCR) has roles in the development of hypothalamic nuclei that regulate energy homeostasis, including the arcuate nucleus [93]. *LYRM7* (ADFI) encodes a mitochondrial complex III assembly factor [94] which is essential for mitochondrial electron transport and oxidative phosphorylation. Variation in *LYRM7* expression or function could affect mitochondrial respiratory efficiency and thereby alter the cellular energy yield per unit of substrate oxidized.

The central nervous system plays a key role in regulating appetite, energy expenditure, and feeding behavior. The identification of multiple neurodevelopmental genes among the feed efficiency candidates underscores the importance of the neuroendocrine axis in shaping feed efficiency phenotypes. *BMP7* (FCR), a member of the TGF-β superfamily, is involved in skeletal development, kidney morphogenesis, brown adipocyte differentiation, and thermogenic regulation [95], but it has also been shown to act on hypothalamic neurons to suppress food intake. *DISC1* (FCR) encodes a multifunctional scaffold protein expressed predominantly in the brain, where it organizes protein complexes involved in neuronal migration, synaptic plasticity, and cAMP signaling [96]. *DISC1* interacts with phosphodiesterase 4B (PDE4B) to regulate intracellular cAMP levels in hypothalamic neurons, a pathway central to leptin and insulin signaling and therefore to the central regulation of feed intake and energy balance. *AUTS2* and *PNKD*, both identified as candidate genes for RFI, have been implicated in neurological functions. *AUTS2* is involved in the regulation of neuronal development and differentiation [97], whereas *PNKD* (also known as myofibrillogenesis regulator 1, MR-1) encodes a presynaptic protein that regulates neurotransmitter release. *TUBB3* (ADFI) is a key protein in neurons and essential as a structural component of microtubules [98]. It is a specific marker used to map enteric neurons and may influence gastrointestinal motility and the secretion of gastrointestinal enzymes. *HINT1* (ADFI) is involved in various functions such as cell signaling and gene expression, and it has been identified as crucial for the peripheral nervous system [99]. Association of *HINT1* with ADFI loci may therefore reflect a peripheral mechanism of feed intake regulation. *PRDM7* (ADFI) is a member of the PRDM family of histone methyltransferases, which are essential in the nervous system for shaping neural identity and, in the musculoskeletal system, for regulating bone homeostasis and muscle fiber-type specification [100]. *ZNF276* (ADFI) is a zinc finger protein; the main function of ZNF in the brain is to promote the development of different parts of the brain and the differentiation of neural stem cells [101]. *ADGRL3* (ADFI) belongs to the adhesion GPCR family and functions as a synaptic organizer in dopaminergic and noradrenergic neurons [102].

*DEF8* (ADFI) displays a predominantly neuronal expression pattern in the central nervous system of murine models [103] and plays a critical role in maintaining cellular homeostasis within the nervous system [104]. *DBNDD1* (ADFI) is a protein-coding gene with significant sequence homology to *DTNBP1* (Dystrobrevin Binding Protein 1), the gene encoding dysbindin. Dysbindin is a multifunctional protein involved in central nervous system development and intracellular transport, contributing to synaptic vesicle biogenesis, dendrite and neurite formation, and the trafficking of glutamate and dopamine receptors [105].

Three candidate genes reflect the relationship between immune activation and feed efficiency; in fact, chronic low-grade inflammation diverts nutrients and energy away from productive processes toward immune maintenance and acute-phase responses [106]. *CXCR2* and its closely related paralog LOC100515345 (both identified for RFI) are involved in neutrophil recruitment to sites of inflammation, acting as a key regulator of the acute-phase response [107]. *FANCA* (ADFI) encodes the largest subunit of the Fanconi anemia (FA) core complex, a key component of the FA pathway, which regulates innate immune signaling [108]. The association of *FANCA* with ADFI may reflect its role in modulating inflammatory tone in intestinal epithelium and the enteric immune system.

### 3.4. Gene Network Analysis

**Figure 2:**
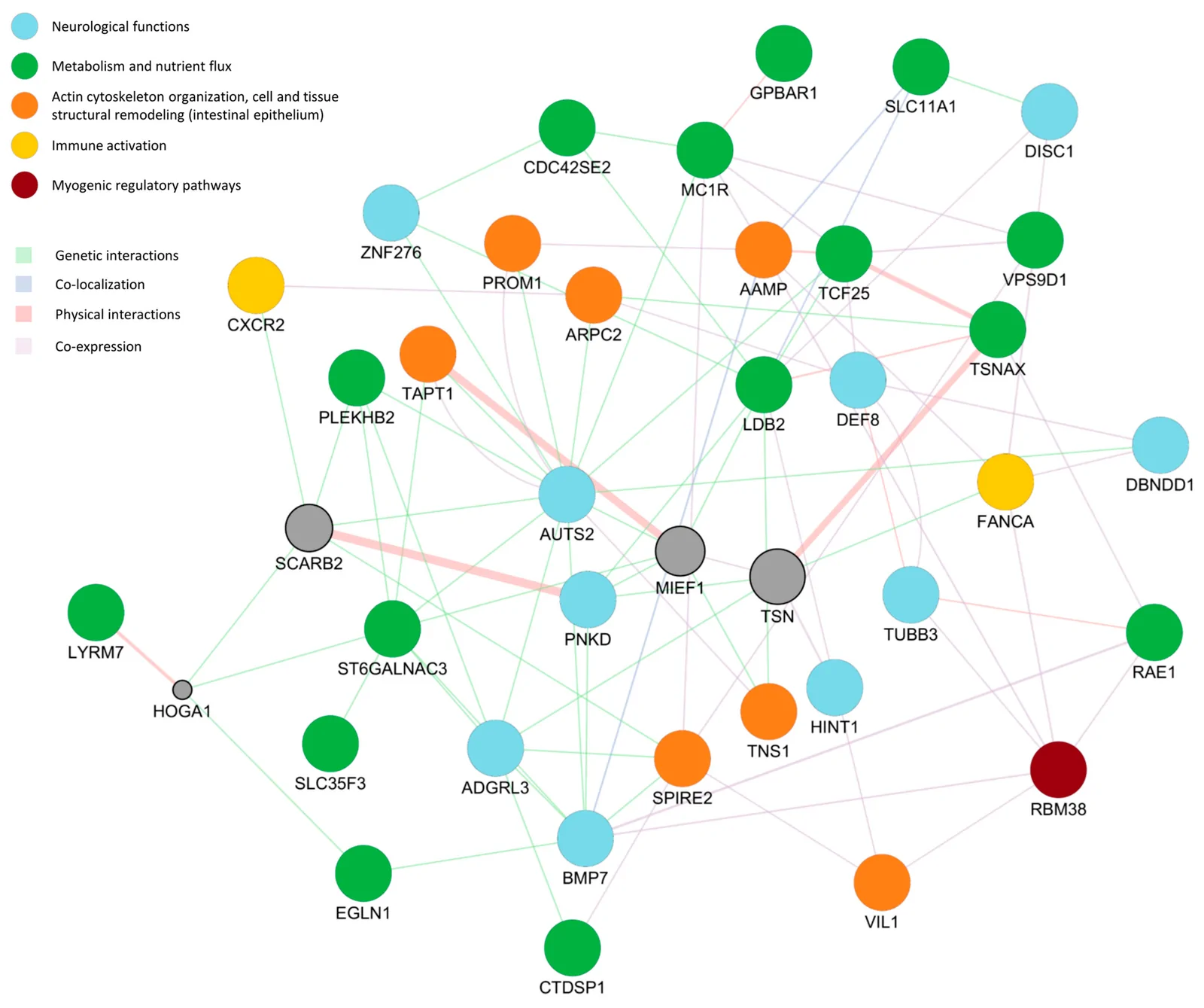
Gene network for feed efficiency traits (residual feed intake, feed conversion ratio) and average daily feed intake. Genes are characterized by colors according to their functions; genes in grey represent predicted or related genes added by GeneMANIA/Cytoscape. Different colors of the lines represent the different interconnections between genes due to co-expression, physical or genetic interactions, or co-localization.

The results of the gene network analysis, integrating available data from diverse sources ranging from individual studies to comprehensive databases of functional interaction networks, are reported in Figure 2. The gene network revealed a highly interconnected set of candidate genes associated with feed efficiency traits (RFI, FCR, and ADFI), suggesting that these phenotypes are regulated by multiple interacting biological pathways. Several genes, including *AUTS2*, *PNKD*, *MIEF1*, and *TSN*, occupied central positions within the network, indicating potential roles as key regulators linking distinct biological processes. *AUTS2* and *PNKD*, both implicated in neuronal function, were extensively connected to genes involved in metabolic regulation and cellular remodeling, supporting the hypothesis that neural control of feeding behavior and energy homeostasis contributes to variation in feed efficiency. The predicted gene *MIEF1* (Mitochondrial Elongation Factor 1) regulates mitochondrial dynamics by controlling mitochondrial fission and fusion, processes that are critical for cellular energy metabolism. Given the central role of mitochondria in energy production and neuronal function, *MIEF1* may represent an important link between the metabolic and neurological pathways identified in the feed efficiency network. The *TSN* gene encodes a multifunctional DNA- and RNA-binding protein involved in RNA metabolism and dendritic mRNA transport, which may explain its extensive connections with genes associated with neurological functions. In addition, *TSN* formed a functional complex, through physical interaction, with *TSNAX*, which regulates the expression of metabolic genes in the hypothalamus and has been implicated in the central regulation of body weight and energy homeostasis.

In the network, a prominent cluster of genes involved in metabolism and nutrient utilization was co-expressed with genes associated with intestinal tissue remodeling and epithelial integrity, highlighting the critical role of the intestinal epithelium in nutrient digestion and absorption, and consequently in determining feed utilization efficiency. The predicted gene *HOGA1* (4-hydroxy-2-oxoglutarate aldolase 1) encodes the mitochondrial enzyme 4-hydroxy-2-oxoglutarate aldolase, which is predominantly expressed in the liver and kidneys and plays an essential role in amino acid catabolism. *HOGA1* was connected through genetic and physical interactions to several genes involved in nutrient metabolism (*LYRM7*, *EGLN1*, and *ST6GALNAC3*), as well as to the predicted gene *SCARB2* (Scavenger Receptor Class B Member 2), an intracellular receptor that mediates lysosomal enzyme transport and participates in lipid and cholesterol trafficking, suggesting a potential link between amino acid metabolism, lysosomal function, and nutrient utilization.

Of particular interest is the co-localization of *AAMP*, *BMP7*, and *SLC11A1*, which may indicate common biological pathways or coordinated regulatory mechanisms associated with feed efficiency. While *AAMP* is involved in endothelial cell migration within the gastrointestinal tract, *BMP7* contributes to the central regulation of feed intake through its action on hypothalamic neurons, and *SLC11A1* plays a role in iron homeostasis and energy extraction from feed substrates. Together, these genes highlight the interplay between gut function, nutrient metabolism, and neuroendocrine regulation in determining feed efficiency.

Collectively, the network architecture suggested that the cited biological pathways do not operate independently but rather form an interconnected regulatory framework that may contribute to complex physiological processes involved in regulation of feed intake and variations in feed efficiency. It should be noted that the GeneMANIA analysis was performed to provide biological context for the identified candidate genes rather than to support the statistical evidence of the GWAS associations. As the network is built from previously reported genetic, physical, and functional interactions, it reflects current biological knowledge and facilitates the interpretation of the identified genes within shared pathways and biological processes. Therefore, the observed gene interactions should be interpreted as complementary biological evidence that helps contextualize the GWAS findings.

## 4. Conclusions

Improving feed efficiency is a key objective in pig production, as it contributes substantially to reducing environmental impact, enhancing farm profitability, and optimizing the use of resources. The present study contributes novel insights by exploring the genetic basis of residual feed intake, feed conversion ratio and daily feed intake, providing a more detailed understanding of the underlying genomic regions influencing feed efficiency traits in heavy pigs.

Our genome-wide association study identified 4, 4, and 8 candidate genomic regions with high posterior probability for the direction of their effects on residual feed intake, feed conversion ratio and average daily feed intake, respectively. SNPs were mainly located on SSC3, 13 and 15 (residual feed intake), SSC8, 14 and 17 (feed conversion ratio), and SSC1, 2, 6, 8 and 11 (average daily feed intake).

The search for candidate genes identified 41 potential genes linked to the traits under investigation. Functional annotation of these genes revealed their involvement in a diverse set of biological mechanisms, primarily related to intestinal epithelial development and maintenance, including tissue renewal, cellular homeostasis, and digestive secretory functions. Additional candidate genes were associated with nutrient utilization and energy metabolism, as well as neural pathways governing appetite control, feed consumption, and satiety regulation.

The results reinforce the likely multifactorial nature of feed efficiency in pigs, highlighting the involvement of a diverse range of biological processes, all of which appear to act in a coordinated manner. This complexity suggests that feed efficiency traits are governed by the interplay of multiple regulatory networks and physiological systems acting simultaneously, highlighting the importance of considering age, physiological stage, and production goals when designing breeding strategies aimed at improving feed efficiency in pigs.

## Figures and Tables

**Table 1:** Descriptive statistics of the traits included in the study.

Trait	Acronym	N	Mean ± SD
Residual feed intake (kg/day)	RFI	201	0.000 ± 0.181
15% lowest (kg/day)		30	−0.287 ± 0.073
15% highest (kg/day)		30	0.272 ± 0.075
Average daily feed intake (kg/day)	ADFI	201	3.176 ± 0.473
15% lowest (kg/day)		30	2.477 ± 0.210
15% highest (kg/day)		30	3.921 ± 0.215
Feed conversion ratio (kg/kg)	FCR	201	3.793 ± 0.296
15% lowest (kg/kg)		30	3.401 ± 0.069
15% highest (kg/kg)		30	4.312 ± 0.206

**Table 2:** Details of the SNPs identified for residual feed intake. *Sus scrofa* chromosome (SSC), position in base pairs (bp), estimate of the regression coefficient and 95% highest posterior density interval (HPD95%), odds ratio (OR) and confidence interval (CI), and probability of the OR (P_OR_) being greater than 1 (for OR > 1) or lower than 1 (for OR < 1) are reported.

SSC	Position (bp)	Estimate (HPD95%)	OR (CI)	P_OR_
3	14,818,366	1.02 (−0.24, 2.30)	2.77 (0.78, 9.94)	0.94
13	186,567,034	−1.25 (−2.68, 0.10)	0.29 (0.07, 1.10)	0.97
15	57,638,216	−1.46 (−2.80, −0.11)	0.23 (0.06, 0.89)	0.99
15	120,284,494	0.70 (−0.62, 2.04)	2.02 (0.54, 7.72)	0.85

**Table 3:** Details of the SNPs identified for feed conversion ratio. *Sus scrofa* chromosome (SSC), position in base pairs (bp), estimate of the regression coefficient and 95% highest posterior density interval (HPD95%), odds ratio (OR) and confidence interval (CI), and probability of the OR (P_OR_) being greater than 1 (for OR > 1) or lower than 1 (for OR < 1) are reported.

SSC	Position (bp)	Estimate (HPD95%)	OR (CI)	P_OR_
8	11,484,078	0.98 (−0.48, 2.48)	2.68 (0.62, 12.00)	0.91
14	57,047,650	−0.89 (−2.61, 0.70)	0.41 (0.07, 2.02)	0.85
14	58,984,594	−0.88 (−2.55, 0.77)	0.41 (0.08, 2.16)	0.85
17	57,705,289	1.71 (0.27, 3.21)	5.54 (1.31, 24.77)	0.99

**Table 4:** Details of the SNPs identified for average daily feed intake. *Sus scrofa* chromosome (SSC), position in base pairs (bp), estimate and 95% highest posterior density interval (HPD95%), odds ratio (OR) and confidence interval (CI), and probability of the OR (P_OR_) being greater than 1 (for OR > 1) or lower than 1 (for OR < 1) are reported.

SSC	Position (bp)	Estimate (HPD95%)	OR (CI)	P_OR_
1	289,692,169	−1.61 (−3.14, −0.12)	0.20 (0.04, 0.88)	0.98
1	290,288,650	−1.08 (−2.50, 0.33)	0.34 (0.08, 1.39)	0.94
1	291,100,552	−1.45 (−2.93, 0.04)	0.23 (0.05, 1.04)	0.97
2	133,459,493	0.95 (−0.57, 2.54)	2.58 (0.56, 12.71)	0.88
6	127,259	0.67 (−0.54, 1.88)	1.95 (0.59, 6.54)	0.86
6	136,762,487	−1.11 (−2.48, 0.18)	0.33 (0.08, 1.20)	0.95
8	60,120,775	−1.28 (−2.57, −0.04)	0.28 (0.08, 0.96)	0.98
11	28,091,154	−0.88 (−2.41, 0.58)	0.42 (0.09, 1.79)	0.88

**Table 5:** Candidate genes for residual feed intake; *Sus scrofa* chromosome (SSC), SNP position used as reference for gene search (bp), gene name and functions are reported.

SSC	Reference SNP Position (bp)	Gene	Name	Function
3	14,818,366	*AUTS2*	Activator of transcription and developmental regulator	Neuronal development and epigenetic gene regulation
15	57,638,216	*PLEKHB2*	Pleckstrin homology domain containing B2	Retrograde membrane trafficking, transport and cell differentiation; RFI
15	12,0284,494	*AAMP*	Angio-associated migratory cell protein	Endothelial tube formation and cell migration; smooth muscle cell migration
15	12,0284,494	*ARPC2*	Actin related protein 2/3 complex subunit	Intestinal epithelial homeostasis and integrity
15	12,0284,494	*CATIP*	Ciliogenesis associated TTC17 interacting protein	Ciliogenesis regulator; interacts with TTC17 in ciliary assembly
15	12,0284,494	*CTDSP1*	CTD small phosphatase 1	Phosphorylation state of RNA polymerase II
15	12,0284,494	*CXCR2*	C-X-C motif chemokine receptor 2	Neutrophil recruitment and inflammatory signaling
15	12,0284,494	*GPBAR1*	G protein-coupled bile acid receptor 1	Bile acid GPCR (TGR5); stimulates GLP-1 secretion and thermogenesis
15	12,0284,494	*LOC100515345*	C-X-C chemokine receptor type 2	CXCR2 paralog; CXC chemokine receptor involved in inflammatory cell trafficking
15	12,0284,494	*PNKD*	PNKD metallo-beta-lactamase domain containing protein	Regulation of neurotransmitter release
15	12,0284,494	*RUFY4*	RUN and FYVE domain containing 4	Nutrient transport, digestive enzyme receptors in intestinal epithelial cells
15	12,0284,494	*SLC11A1*	Solute carrier family 11-member 1	Iron/zinc homeostasis
15	12,0284,494	*TMBIM1*	Transmembrane BAX inhibitor motif containing 1	Metabolic homeostasis
15	12,0284,494	*TNS1*	Tensin 1	Cell migration, mechanosensing and tissue remodeling
15	12,0284,494	*VIL1*	Villin 1	Microvilli structure and intestinal absorption

**Table 6:** Candidate genes for feed conversion ratio; *Sus scrofa* chromosome (SSC), SNP position used as reference for gene search (bp), gene name and functions are reported.

SSC	Reference SNP Position (bp)	Gene	Name	Function
8	11,484,078	*LDB2*	LIM domain binding 2	Hypothalamic nuclei development and energy homeostasis
8	11,484,078	*PROM1*	Prominin 1	Intestinal/hematopoietic stem cell marker; epithelial renewal and absorptive surface
8	11,484,078	*TAPT1*	Transmembrane anterior–posterior transformation 1	Mediator of intracellular trafficking, primary cilium formation, BMP signaling
14	57,047,650	*SLC35F3*	Solute carrier family 35 member F3	Cellular glucose metabolism, carbohydrate metabolism, energy production
14	58,984,594	*DISC1*	DISC1 scaffold protein	Neuronal scaffold; organizes PDE4B/cAMP complexes in hypothalamic appetite circuits
14	58,984,594	*EGLN1*	EGL-9 family hypoxia inducible factor 1	Oxygen sensing and glycolytic-oxidative metabolic switch
14	58,984,594	*TSNAX*	Translin associated factor X	RNA silencing and hypothalamic metabolic gene regulation
17	57,705,289	*BMP7*	Bone morphogenetic protein 7	Brown adipogenesis, thermogenesis, hypothalamic appetite suppression
17	57,705,289	*RAE1*	Ribonucleic acid export 1	mRNA nuclear export factor; cytoplasmic delivery of metabolic enzyme transcripts
17	57,705,289	*RBM38*	RNA binding motif protein 38	Regulation of cell proliferation, apoptosis, mRNA processing

**Table 7:** Candidate genes for average daily feed intake; *Sus scrofa* chromosome (SSC), SNP position used as reference for gene search (bp), gene name and functions are reported.

SSC	Reference SNP Position (bp)	Gene	Name	Function
2	133,459,493	*CDC42SE2*	CDC42 small effector 2	CDC42 effector; enteroendocrine hormone vesicle secretion
2	133,459,493	*HINT1*	Histidine triad nucleotide binding protein 1	Peripheral nervous system
2	133,459,493	*LYRM7*	LYR motif containing 7	Mitochondrial complex III assembly factor; oxidative phosphorylation efficiency
6	127,259	*CENPBD1*	Putative CENPB DNA-binding domain-containing protein 1	CENP-B DNA-binding domain protein; centromere/chromatin organization
6	127,259	*DBNDD1*	Dysbindin domain containing 1	Central nervous system development and intracellular transport
6	127,259	*DEF8*	Differentially expressed in FDCP 8 homolog	Cellular homeostasis in the nervous system
6	127,259	*FANCA*	FA complementation group A	Innate immune signaling
6	127,259	*MC1R*	Melanocortin 1 receptor	Pigmentation, energy metabolism
6	127,259	*PRDM7*	PR/SET domain 7	Neural identity, bone homeostasis, muscle fiber-type specification
6	127,259	*SPIRE2*	Spire type actin nucleation factor 2	Unbranched filament formation and organelle positioning; affect secretory function in the digestive tract
6	127,259	*TCF25*	Transcription factor 25	bHLH transcription factor; regulates lipid metabolism genes in liver/gut
6	127,259	*TUBB3*	Tubulin beta 3 class III	Neuron-enriched beta-tubulin; microtubule structure in enteric neurons and axons
6	127,259	*VPS9D1*	VPS9 domain containing 1	Rab5 GEF; early endosome fusion and hormone receptor recycling
6	127,259	*ZNF276*	Zinc finger protein 276	Development of different parts of the brain; differentiation of neural stem cells
6	136,762,487	*ST6GALNAC3*	ST6 N-acetylgalactosaminide alpha-2,6-sialyltransferase 3	Golgi sialyltransferase; O-glycan sialylation of mucins and digestive enzymes
8	60,120,775	*ADGRL3*	Adhesion G protein-coupled receptor L3	Synaptic organizer in dopaminergic/noradrenergic neurons

## Data Availability

Restrictions apply to the availability of these data. Data were obtained from animals belonging to Gorzagri (Fonzaso, Italy) and are available from the authors with the permission of Gorzagri.

## Supplementary Materials

The following supporting information can be downloaded at: https://www.mdpi.com/article/10.3390/ani16172662/s1. Figure S1: mean posterior estimates and 95% highest posterior density (HPD95%) intervals for the 50 SNPs selected for RFI based on their Bayes factors calculated in Step 1 and subsequently included in Step 2 of the analysis; Figure S2: mean posterior estimates and 95% highest posterior density (HPD95%) intervals for the 50 SNPs selected for FCR based on their Bayes factors calculated in Step 1 and subsequently included in Step 2 of the analysis; Figure S3: mean posterior estimates and 95% highest posterior density (HPD95%) intervals for the 50 SNPs selected for ADFI based on their Bayes factors calculated in Step 1 and subsequently included in Step 2 of the analysis.

## Abbreviations

The following abbreviations are used in this manuscript:

ADFI	Average daily feed intake
BF	Backfat thickness
BL	Body lipid mass
BL_ADG_	Average daily gain in body lipid
BP	Body protein mass
BP_ADG_	Average daily gain in body protein
BW	Body weight
CI	Confidence interval
EBW	Empty body weight
EBW_MID_	Empty body weight at the midpoint
FCR	Feed conversion ratio
GWAS	Genome-wide association study
HPD95%	95% highest posterior density interval
OR	Odds ratio
PDO	Protected Designation of Origin
P_OR_	Posterior probability of the OR being greater or lower than 1
QTL	Quantitative trait locus
RFI	Residual feed intake
SNP	Single nucleotide polymorphism
SSC	*Sus scrofa* chromosome
