# Supplementary material for: Bayesian Genome-Wide Association Study of Feed Efficiency Traits in Pigs
Source: Animals (Basel). 2026 Aug 25;16(17):2662. doi: 10.3390/ani16172662 (PMC13564335; doi:10.3390/ani16172662)
Supplement: Supplementary file 1 [file animals-16-02662-s001.zip › animals-4436040-supplementary.pdf]

Supplementary material

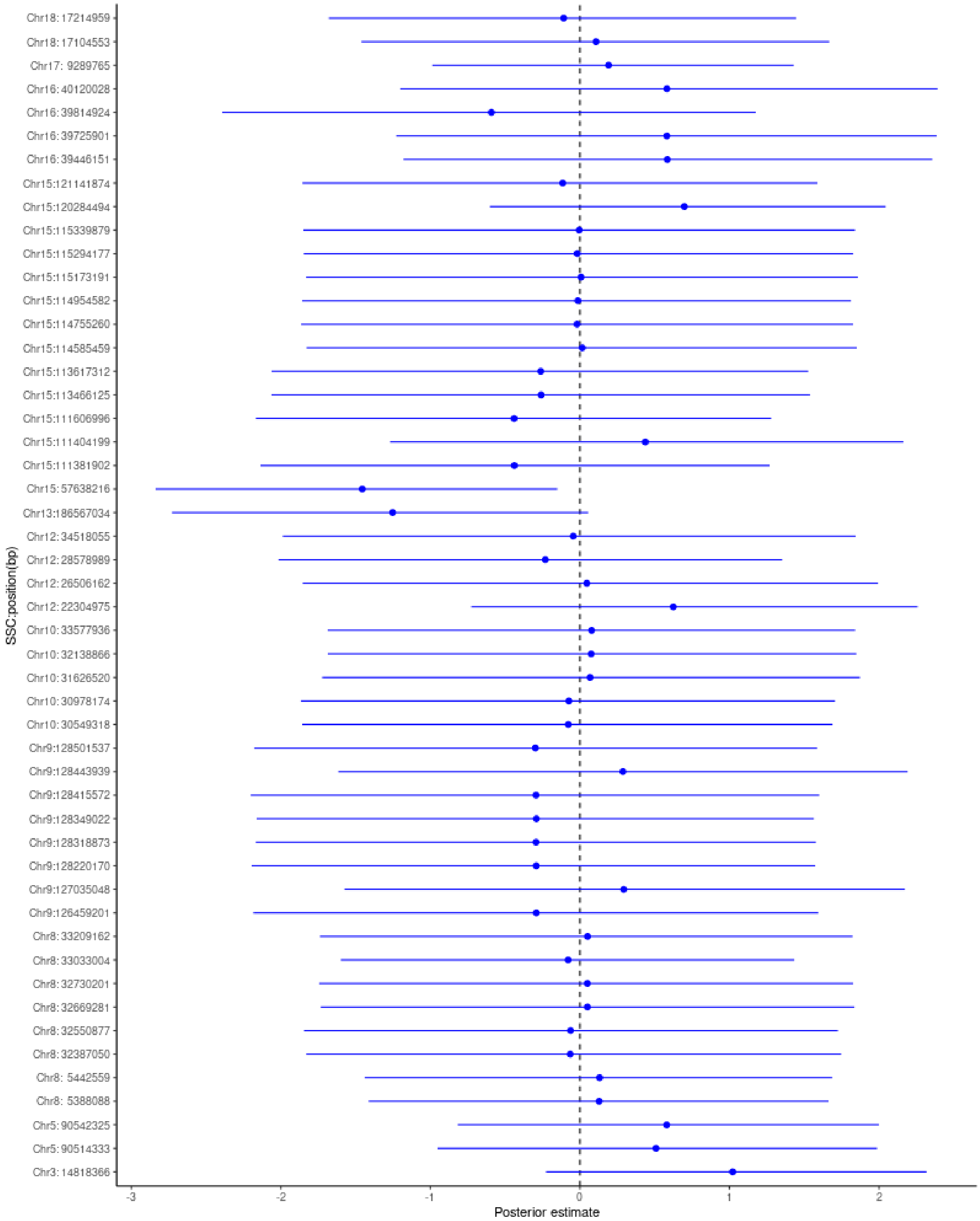

Figure S1. Mean posterior estimates and 95% highest posterior density (HPD95%) intervals for the 50 SNPs selected for RFI based on their Bayes factors calculated in Step 1 and subsequently included in Step 2 of the analysis.

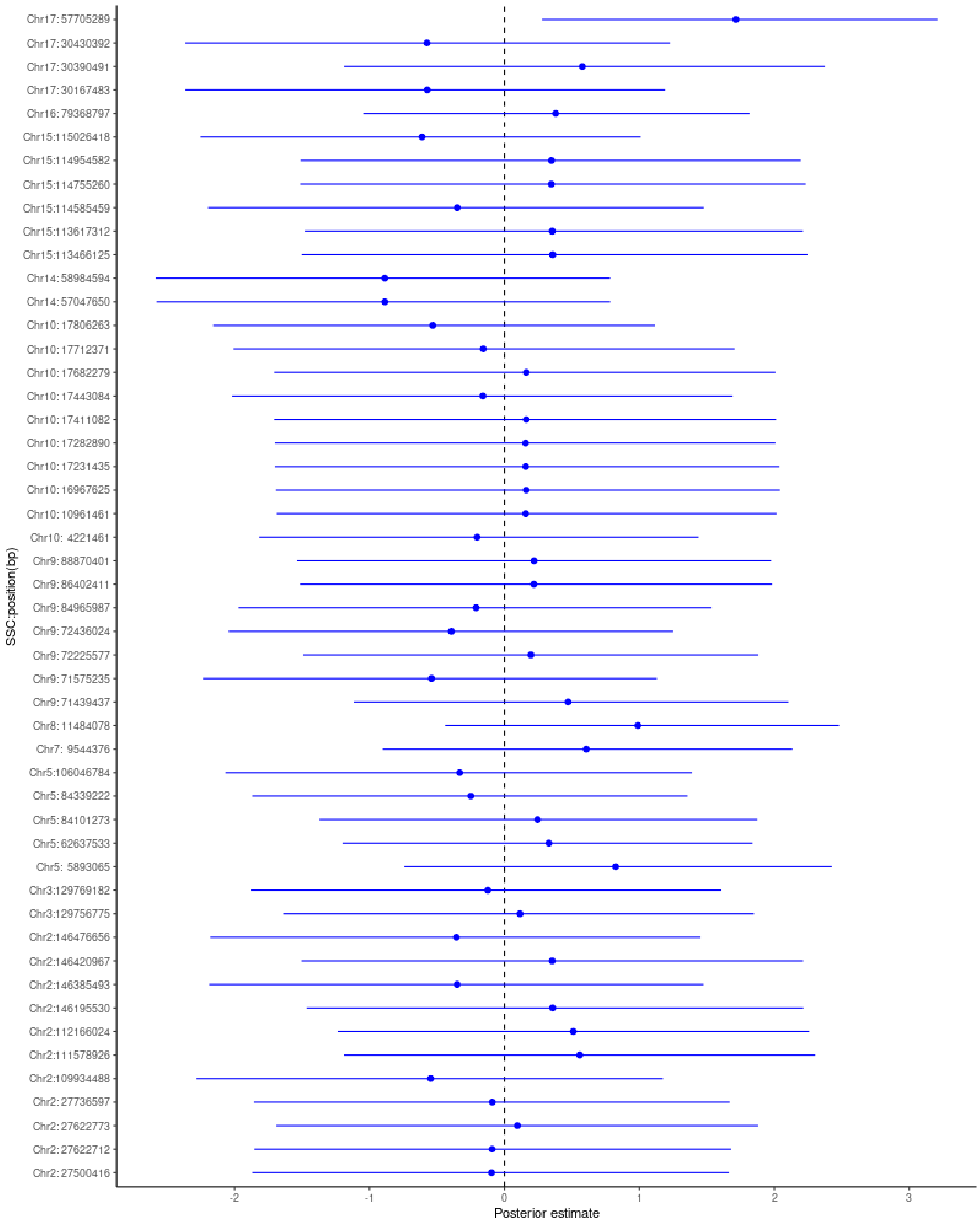

Figure S2. Mean posterior estimates and 95% highest posterior density (HPD95%) intervals for the 50 SNPs selected for FCR based on their Bayes factors calculated in Step 1 and subsequently included in Step 2 of the analysis.

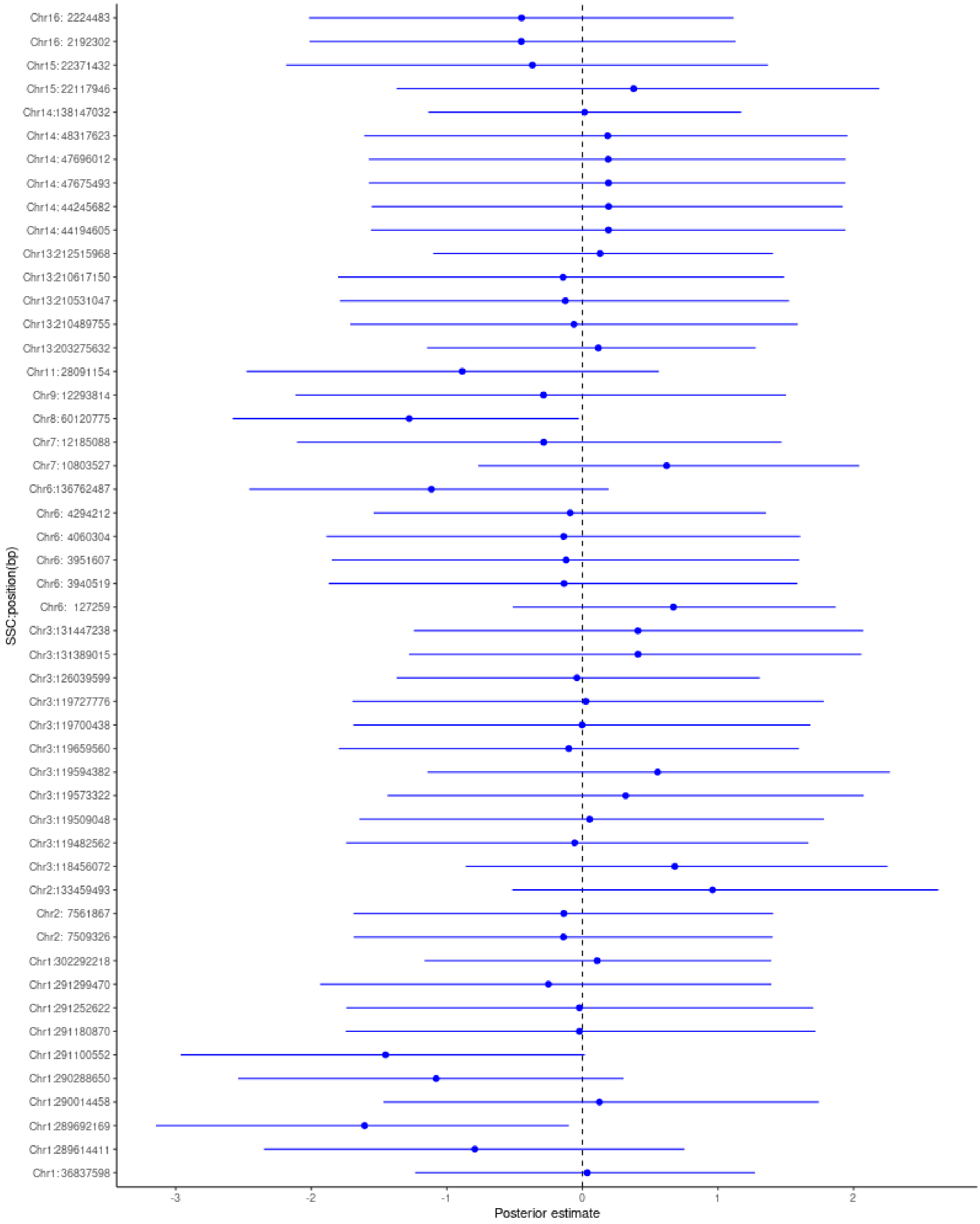

Figure S3. Mean posterior estimates and 95% highest posterior density (HPD95%) intervals for the 50 SNPs selected for ADFI based on their Bayes factors calculated in Step 1 and subsequently included in Step 2 of the analysis.
